# Nine Different Chemical Species and Action Mechanisms of Pancreatic Lipase Ligands Screened Out from *Forsythia suspensa* Leaves All at One Time

**DOI:** 10.3390/molecules22050795

**Published:** 2017-05-12

**Authors:** Tinggui Chen, Yayun Li, Liwei Zhang

**Affiliations:** Institute of Molecular Science, Key Laboratory of Chemical Biology and Molecular Engineering of Ministry of Education, Shanxi University, Taiyuan 030006, China; lisf@sxu.edu.cn (Y.L.); lwzhang@sxu.edu.cn (L.Z.)

**Keywords:** *Forsythia suspensa* leaves, affinity screening, pancreatic lipase inhibitor, obesity

## Abstract

It is difficult to screen out as many active components as possible from natural plants all at one time. In this study, subfractions of *Forsythia suspensa* leaves were firstly prepared; then, their inhibitive abilities on pancreatic lipase were tested; finally, the highest inhibiting subfraction was screened by self-made immobilized pancreatic lipase. Results showed that nine ligands, including eight inhibitors and one promotor, were screened out all at one time. They were three flavonoids (rutin, IC_50_: 149 ± 6.0 μmol/L; hesperidin, 52.4 μmol/L; kaempferol-3-*O*-rutinoside, isolated from *F. suspensa* leaves for the first time, IC_50_ notably reached 2.9 ± 0.5 μmol/L), two polyphenols (chlorogenic acid, 3150 ± 120 μmol/L; caffeic acid, 1394 ± 52 μmol/L), two lignans (phillyrin, promoter; arctigenin, 2129 ± 10.5 μmol/L), and two phenethyl alcohol (forsythiaside A, 2155 ± 8.5 μmol/L; its isomer). Their action mechanisms included competitive inhibition, competitive promotion, noncompetitive inhibition, and uncompetitive inhibition. In sum, using the appropriate methods, more active ingredients can be simply and quickly screened out all at one time from a complex natural product system. In addition, *F. suspensa* leaves contain numerous inhibitors of pancreatic lipase.

## 1. Introduction

Obesity has become one of the greatest threats to human health in this century. It is recognized as the largest global chronic disease by WHO [1,2]. At least 3,400,000 adults die each year as a result of being overweight or obese [3,4]. Because pancreatic lipase (PL) can decompose 50–70% of fat, developing its inhibitor as an anti-obesity drug is preferred. Orlistat is a type of long-term PL inhibitor found and modified from *Streptomyces toxytricini* and is currently one of the main drugs for treating obesity. However, this drug produces several side effects including fatty diarrhea, stool urgency, fecal incontinence, allergies, and liver function damage [5,6,7].

In recent years, developing safer and more effective PL inhibitors from natural compounds has attracted more and more attention [8,9,10,11], and some new technologies are emerging [12,13]. We also used immobilized enzyme technology to screen PL inhibitors from *Polygonum cuspidatum* [14].

*Forsythia suspensa* (Thunb.) Vahl is widely distributed in China, Japan, and Korea. Their fruits have anti-inflammatory, anti-viral, anti-pyretic, anti-liver injury, and other effects [15,16,17,18]. Their leaves are often used for tea. In this study, in order to find out whether *F. suspensa* tea, like black tea [19], oolong tea [20], and green tea [21], also possess an inhibitory effect on PL, its subfractions were prepared, and their inhibitive ability on PL were detected; then, the highest inhibiting subfraction was screened by self-made immobilized PL.

## 2. Results and Discussion

### 2.1. Inhibition of the Subfractions of F. suspensa Leaves on PL

As a positive drug, the inhibition rate of 5 μg/mL orlistat reached 58%, and its success proved the feasibility of the enzyme activity assay. The inhibitory effects of crude extracts and various extract subfractions of *F. suspensa* leaves on PL at 1500 μg/mL are shown in Table 1. The table shows that the inhibition rate of the remaining parts on PL was the highest and reached 81.48%. Thus, the remaining precipitation of *F. suspensa* leaves was selected as the screening object of immobilized enzyme.

### 2.2. Screening of PL Inhibitors in the Remaining Precipitation

**Figure 1:**
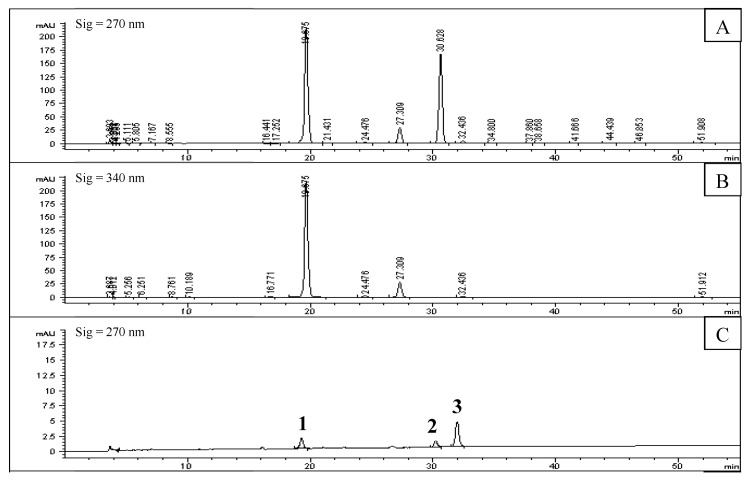

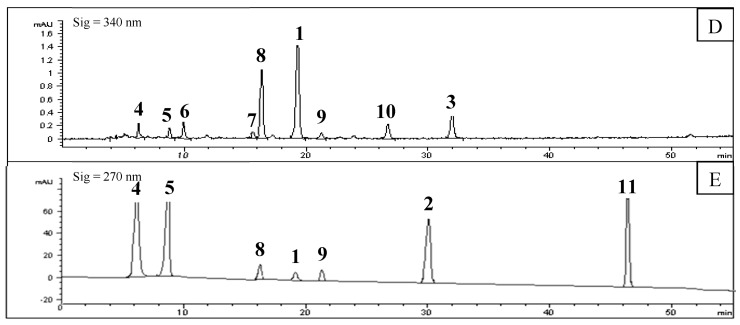
HPLC chromatograms of remaining precipitate of *F. suspensa* leaves (**A**: 270 nm; **B**: 340 nm), solution of screened out compounds (**C**: 270 nm; **D**: 340 nm), and mixed standard substances (**E**: 270 nm); 7 standard substances were completely detected at 270 nm, so 340 nm was not done) (**1**: Rutin; **2**: Phillyrin; **4**: Chlorogenic acid; **5**: Caffeic acid; **8**: Forsythiaside A; **9**: Hesperidin; **11**: Phillygenin).

The HPLC results are shown in Figure 1. Figure 1A,B shows that 21 absorption peaks occurred in the remaining precipitation. Ten compounds, which may interact with PL, were screened out by the immobilized enzyme (Figure 1C,D).

**Figure 2:**
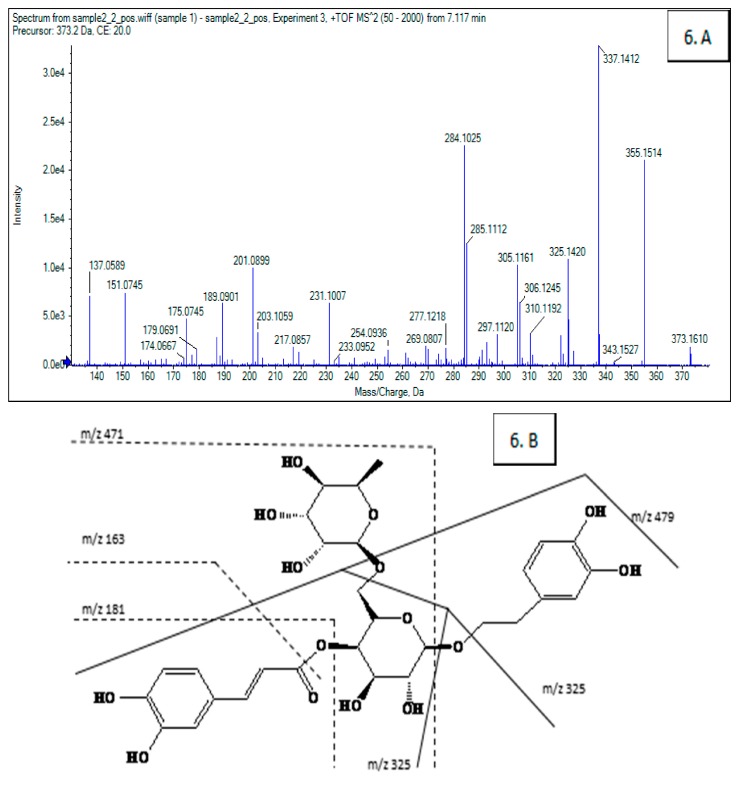

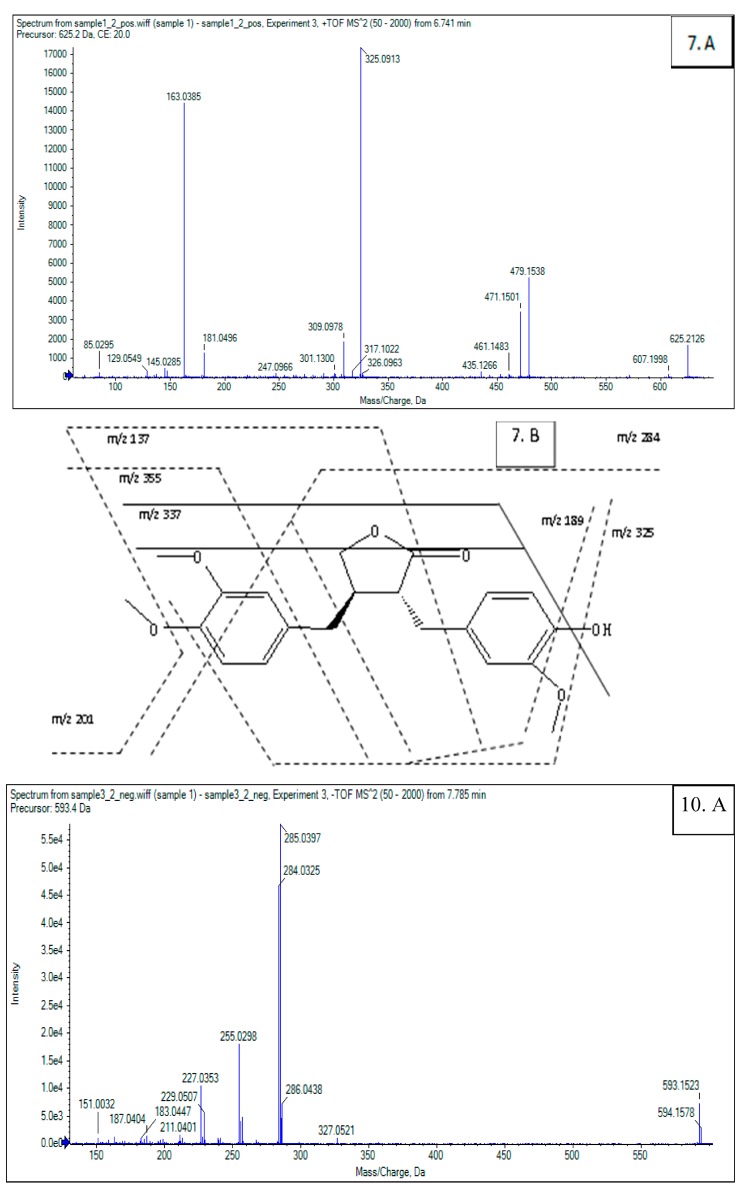

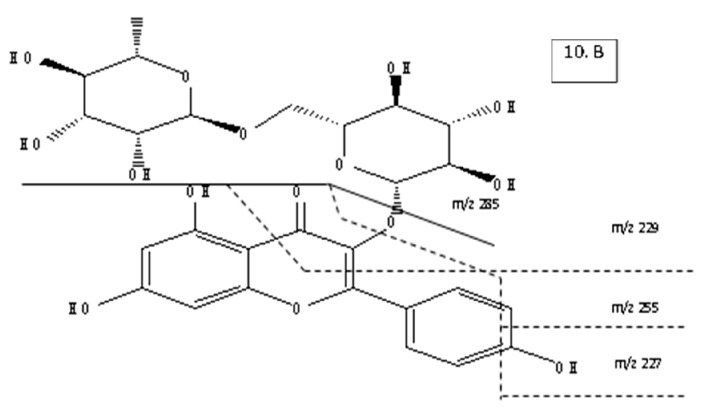
MS2 spectra (**A**) and fragmentation schemes (**B**) of Compounds **6**, **7**, and **10** of *F. suspensa* leaves. (**6**: Isomer of forsythiaside A; **7**: Arctigenin; **10**: Kaempferol-3-*O*-rutinose).

**Figure 3:**
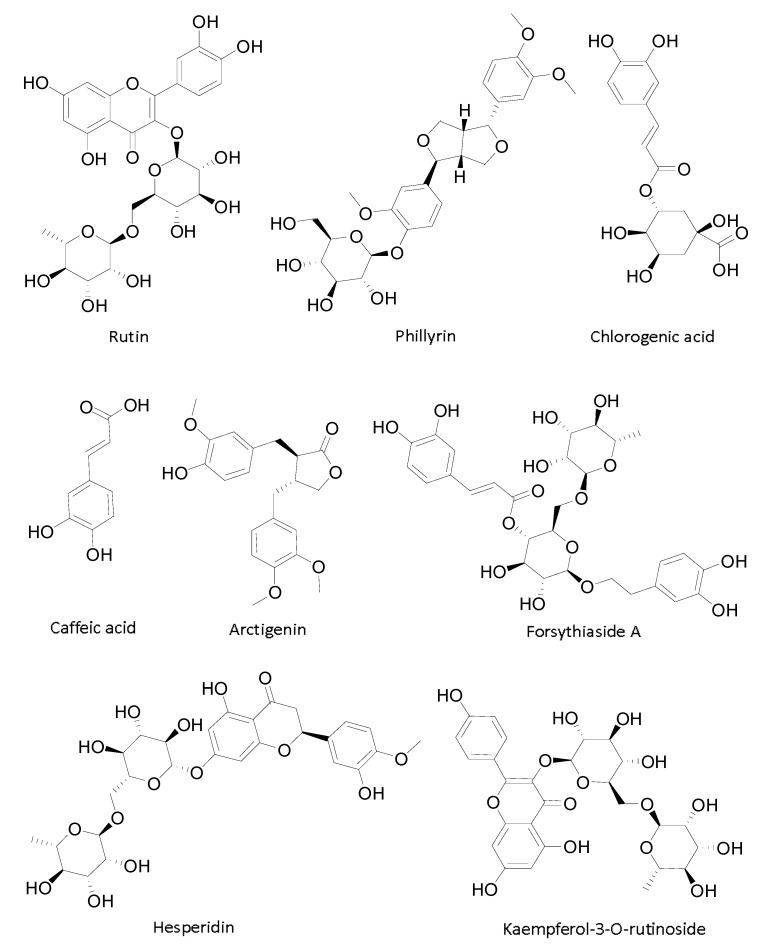
Tentative identified 8 PL ligands from *F. suspensa* leaves: Rutin (**1**); Phillyrin (**2**); Chlorogenic acid (**4**); Caffeic acid (**5**); Arctigenin (**7**); Forsythiaside A (**8**); Hesperidin (**9**); Kaempferol-3-*O*-rutinoside (**10**).

Among the ten compounds, Compounds **1**, **2**, **4**, **5**, **8** and **9** were rutin, phillyrin, chlorogenic acid, caffeic acid, forsythiaside A, and hesperidin respectively by compared with the mixed standard substances (Figure 1E). The undetermined compounds were separated and identified by HPLC-MS/MS; Compounds **6**, **7,** and **10** were identified as an isomer of forsythiaside A, arctigenin, and kaempferol-3-*O*-rutinoside, shown in Figure 2 and Table 2. Compound **3** had not been previously identified. The fully identified 8 PL ligands from *F. suspensa* leaves are shown in Figure 3.

### 2.3. The Detection of Enzyme Inhibitory Activity and Competition Types of All Inhibitors

**Figure 4:**
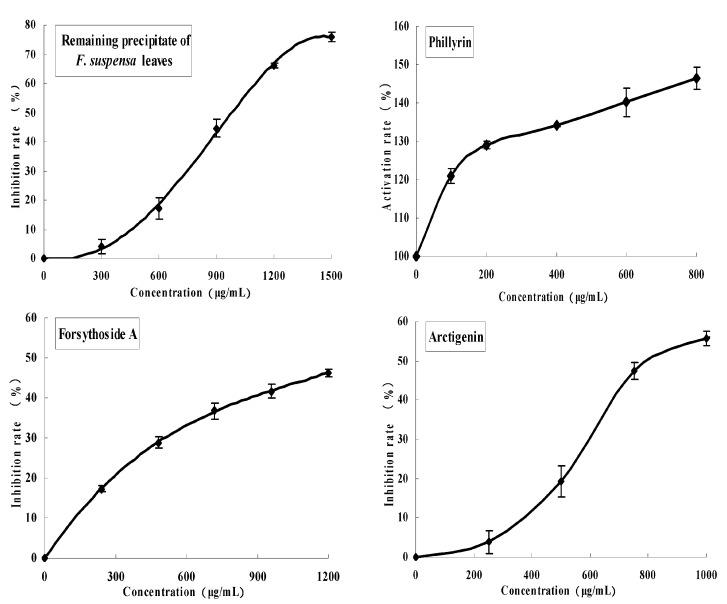
Inhibitive effects of remaining precipitate, phillyrin (Compound **2**), forsythioside A (Compound **8**), and arctigenin (Compound **7**) of *F. suspensa* leaves on pancreatic lipase.

Of the eight compounds with identified structures, references [22,23,24,25] have shown the PL inhibitory activities of five compounds, including **4** (chlorogenic acid), **5** (caffeic acid), **1** (rutin), **9** (hesperidin), and **10** (kaempferol-3-*O*-rutinoside) (their inhibitory abilities were not tested in this study; these references only illustrated the success of this experiment system). The PL inhibitory activities of Compounds **7** (arctigenin), **8** (forsythiaside A), and **2** (phillyrin) were detected. The results are shown in Figure 4. Of these eight compounds, the inhibitory effect of kaempferol-3-*O*-rutinoside on PL was the strongest, with an IC_50_ value of 2.9 ± 0.5 μmol/L, followed by hesperidin and rutin, with IC_50_ values of 52.4 and 149 ± 6.0 μmol/L, respectively. The inhibitory effects of caffeic acid, arctigenin, forsythiaside A, and chlorogenic acid were lower. Interestingly, phillyrin exhibited no inhibitory effect on PL, but showed a promoting effect (see Table 3).

**Figure 5:**
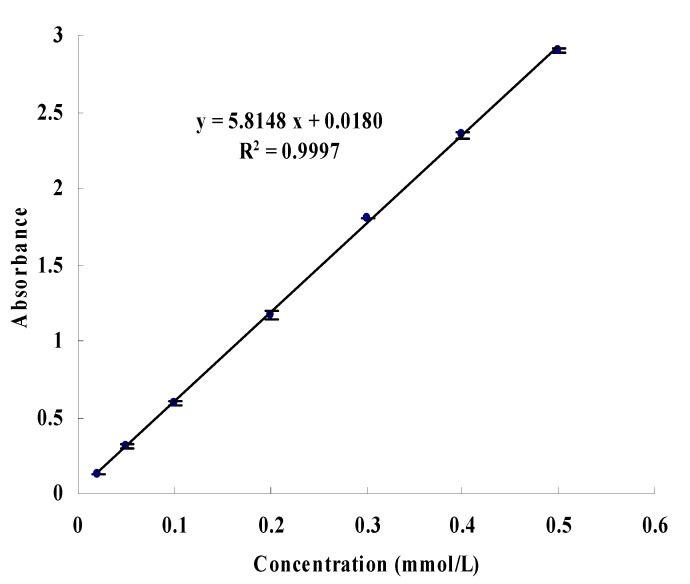
Standard curve of *p*-nitrophenol.

**Figure 6:**
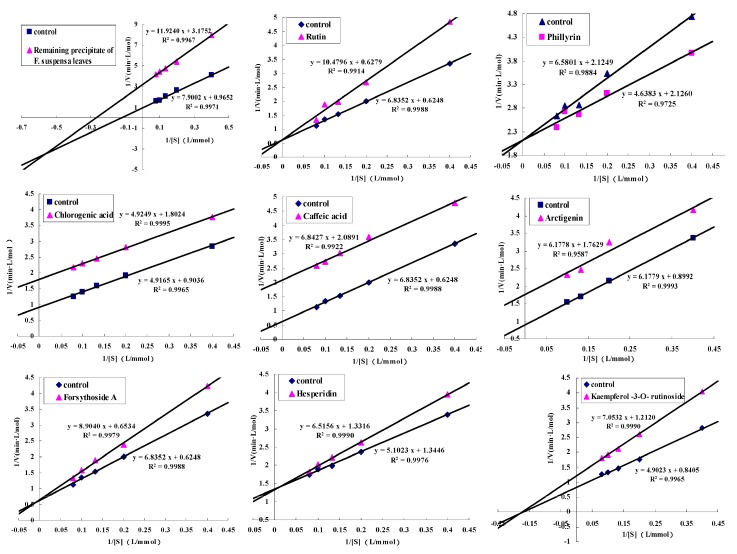
Inhibitive modes of remaining precipitate and screened compounds on pancreatic lipase in *F. suspensa* leaves (remaining precipitate, Rutin (**1**), Phillyrin (**2**), Chlorogenic acid (**4**), Caffeic acid (**5**), Arctigenin (**7**), Forsythiaside A (**8**), Hesperidin (**9**), Kaempferol-3-*O*-rutinoside (**10**)).

The standard curve of pNP is shown in Figure 5, the inhibition types of the remaining precipitation and the eight compounds were both detected and are shown in Figure 6. Kaempferol-3-*O*-rutinoside showed non-competitive inhibition. Rutin, hesperidin, and forsythiaside A showed competitive inhibition (the isomer of forsythiaside A might have the same ability). Caffeic acid, chlorogenic acid, and arctigenin exhibited uncompetitive inhibition. Phillyrin was a special compound that showed a competitive promoting effect.

All of the experimental results of the nine ligands are shown in Table 3.

### 2.4. Discussion

In this study, nine ligands that could interact with PL were screened out all at one time by an immobilized enzyme screening model. The reasons are listed as follows: (1) We chose an immobilized enzyme carrier that has as little adsorption ability as possible to avoid nonspecific adsorption; (2) We closed the remaining carboxyl of the magnetic bead surface via small- and little-absorption-ability compounds; (3) We screened for a higher active subfraction; (4) We chose a suitable eluent and denaturant, and their concentration; (5) We chose a suitable HPLC mobile phase and elution gradient to obtain a smooth baseline and good separation effects; (6) We scanned a full wavelength of the screened sample and then chose suitable HPLC detection wavelengths. Put simply, using these methods can lead to compounds with less nonspecific absorption and with more specific absorption.

In this study, two new PL inhibitors, arctigenin and forsythiaside A, were found. Kaempferol-3-*O*-rutinoside, which could not be found from *F. suspensa* before, was screened out for the first time, and its IC_50_ notably reached 2.9 ± 0.5 μmol/L; moreover, phillyrin, as a promotor of PL was also found. The isomer of forsythiaside A and compound **6** need to be isolated and identified.

## 3. Materials and Methods

### 3.1. Materials

The following materials were acquired and used: Orlistat (Chongqing Pharscin Pharmaceutical Group Co., Ltd., Chongqing, China); carboxyl-terminated magnetic beads (10 mg/mL, 1 μm) (Allrun, Shanghai, China); *F. suspensa* leaves (Shanxi University campus, Taiyuan, China); PL, *N*-3-dimethylaminopropyl-*N*′-ethyl-carbodiimide HCl (EDC), *N*-hydroxysuccinimide (NHS), 2-(*N*-morpholino) ethanesulfonic acid (MES), Tween 20, Tris and glycine (Gly) (Solarbio, Beijing, China); *p*-nitrophenyl butyrate (*p*NPB), and *p*-nitrophenol (*p*NP) (Sigma-Aldrich, St. Louis, MO, USA); standard compounds, including chlorogenic acid, caffeic acid, forsythiaside A, rutin, hesperidin, and phillyrin (Must Bio-Technology Co., Ltd., Sichuan, China); HPLC-grade methanol and 96-well microtiter plates (Fisher Scientific, Shanghai, China). Other reagents were analytically pure. All solutions were prepared by ultra pure water.

### 3.2. Preparation of the Subfractions of F. suspensa Leaves

The leaves of *F. suspensa* (Thunb.) Vahl were collected in the campus of Shanxi University in July 2014 and identified by Prof. Liwei Zhang, Institute of Molecular Science, Shanxi University, Taiyuan 030006, China. A voucher specimen (no. SXTY-05337) had been deposited at the Herbarium of Institute of Molecular Science, Shanxi University, China. First, the dried leaf powder (300 g) was added to 3 L of 70% ethanol, reflux was extracted for 1 h, and then the hot extraction solution was filtered. The extraction and filtration process was repeated twice. Second, the filtrates were concentrated to 500 mL (note: there was some water in 500 mL of concentrated liquid; if it was dried, its weight was 99.29 g, and the yield was 33.09%). Third, petroleum ether and ethyl acetate was used for extraction sequentially (1:1, *v*/*v*, twice) from the cooled concentrated liquid. Fourth, ethanol and water were employed to dissolve the remaining mixture sequentially (1:10, *v*/*v*, once) (note: due to a long extraction time, repeated extraction, and hot filtration, the 70% ethanol extraction contained various substances that could not be completely dissolved in cold ethanol and could be divided into five parts: an easily soluble part in cold petroleum ether, an easily soluble part in cold ethyl acetate, a soluble part in cold ethanol, a soluble part in cold water, and a final water-insoluble part). Finally, the corresponding subfractions and the final water-insoluble substance were dried. The petroleum ether extraction, ethyl acetate extraction, ethanol-soluble substance, water-soluble substance, and the final water-insoluble substance yields were 4.46%, 9.13%, 15.04%, 2.43%, and 1.81%, respectively.

### 3.3. Determination of Enzyme Inhibitory Activity of All Subfractions

A PL solution (100 µL, 0.5 mg/mL) was placed in a 1.5 mL centrifuge tube, and 20 µL of sample solution was then added. A Tris–HCl buffer (pH 7.5) was added to 900 µL, mixed, and incubated for 15 min at 37 °C. A *p*NPB solution (100 µL, 10 mmol/L) was added to the solution, which was oscillated and then quickly transferred to a 96-well microtiter plate. Finally, the change rate K, which is the change of absorbance of the solution at 400 nm over 15 min, was measured with a microplate reader. The inhibition rate was calculated as follows:

Inhibition rate (%) = (K normal value − K experimental value)/K normal value × 100%.

In the reaction system, PL, *p*NPB, and DMSO were used as a normal group, PL, *p*NPB, and orlistat (DMSO solution) as a positive control group, and PL, *p*NPB, and inhibitor (DMSO solution) as an experimental group. K normal value = (*p*NP absorbance (after 15 min) − *p*NP absorbance (0 min))/15, namely the rate of enzyme reaction, in normal group at 400 nm, and K experimental value was the same, but in experimental group. *p*NP was the product of *p*NPB catalyzed by PL. 0 min meant the beginning of absorbance detection by Microplate Reader. Each experiment was repeated three times.

The inhibition rates of all subfractions on PL were measured at 1.5 mg/mL.

### 3.4. Screening of Inhibitors in the Subfraction with the Highest Inhibitory Activity

Synthesis, characterization, stability, specificity, and repeatability of immobilized pancreatic lipase were published in [14]. 

The subfraction with the highest inhibitory activity was used to screen the PL inhibitors. The process was similar to the validation of immobilized PL. A Thermo Scientific Hypersil Gold C18 column (4.6 mm × 250 mm, 5 μm) was used for HPLC separation and detection. The detection wavelength was 270 and 340 nm. The column temperature and flow rate were 25 °C and 0.8 mL/min, respectively. The mobile phase was composed of methanol (A) and 0.3% acetic acid solution (B). The gradient elution was set as follows: 0–8 min, 30–33% A; 8–24 min, 33–40% A; 24–39 min, 40–48% A; and 39–55 min, 48–64% A.

### 3.5. Identification of Screened-Out Compounds

Comparison with the HPLC chromatograms of a mixture of standard compounds: Suitable amounts of chlorogenic acid, caffeic acid, forsythiaside A, rutin, hesperidin, phillyrin, and forsythiaside standards were dissolved in methanol and detected according to the aforementioned chromatographic conditions.

LC-MS identification: The compounds that were not matched with HPLC chromatograms of a mixture of standard compounds were separated and tentatively identified by LC-MS.

### 3.6. Determination of Enzyme Inhibitory Activity and Competition Types of Screened-Out Compounds

The detection method of enzyme inhibitory activity was similar to a determination of enzyme inhibitory activity of all subfractions. The test method of competition types was that *p*NP standard solution with concentrations of 0.02, 0.05, 0.1, 0.2, 0.3, 0.4, and 0.5 mmol/L was precisely prepared. When the standard curve was drawn, a PL solution (0.5 mg/mL) and the inhibitor solution with concentrations close to IC_50_ were prepared. The solution with no inhibitor was regarded as the control group, and solutions with an addition of inhibitor was regarded as the experimental group. The enzyme reaction rates were determined when the substrate concentrations were 0.25, 0.50, 0.75, 1.00, and 1.25 mmol/L. The map was drawn by Lineweaver–Burk double reciprocal plot method.

## 4. Conclusions

Using the appropriate methods, more active ingredients can be simply and quickly screened out all at one time from a complex natural product system. In conclusion, *F. suspensa* leaves contain numerous inhibitors of pancreatic lipase. A potential use of this tea for weight loss remains to be shown in pharmacological studies.

## Figures and Tables

**Table 1:** Inhibitory ratio of subfractions of *F. suspensa* leaves on pancreatic lipase (*n* = 3).

Subfractions (1500 μg/mL)	Inhibitory Ratio
Crude extract	39.28%
Acetic ether extract	44.17%
Ethanol extract	15.91%
Water extract	50.21%
Remaining precipitate	81.48%

**Table 2:** MS2 analysis of Compounds **6**, **7**, and **10** of *F. suspensa* leaves.

Peak No.	*t*_R_ (min)	UV (nm)	MS (*m*/*z*)	MS2 (*m*/*z*)	Tentative Identification
6	9.986	216,247,290,330	624	479, 471, 325, 309, 181, 163	Isomer of forsythiaside A
7	15.682	208,230,280	372	355, 337, 325, 284, 201, 189, 137	Arctigenin
10	26.716	265,337	594	285, 255, 229, 227,	Kaempferol-3-*O*-rutinose

**Table 3:** Nine pancreatic lipase ligands screened out from *F. suspensa* leaves (*n* = 3).

No.	Compounds	Species	IC_50_ (μg/mL)	IC_50_ (μmol/L)	Mechanisms
1	Rutin [22]	Flavonoids	91 ± 3.66 *	149 ± 6.0 *	competitive inhibition
2	Phillyrin	Lignanoids	Promotion	Promotion	promotor
3	Isomer of forsythiaside A	Phenethylols	No tested	No tested	possible competitive inhibition
4	Chlorogenic acid [23]	Polyphenols	1120 ± 40 *	3150 ± 120 *	uncompetitive inhibition
5	Caffeic acid [24]	Polyphenols	251.2 ± 9.3 *	1394 ± 52 *	uncompetitive inhibition
7	Arctigenin	Lignanoids	793.4 ± 3.9	2129 ± 10.5	uncompetitive inhibition
8	Forsythiaside A	Phenethylols	1346 ± 5.3	2155 ± 8.5	competitive inhibition
9	Hesperidin [25]	Flavonoids	32 *	52.4 *	competitive inhibition
10	Kaempferol-3-*O*-rutinoside [22]	Flavonoids	1.7 ± 0.3 *	2.9 ± 0.5 *	noncompetitive inhibition

Note: “*” represented that the data came from references.
